# Fruit and Vegetable Peel-Enriched Functional Foods: Potential Avenues and Health Perspectives

**DOI:** 10.1155/2022/8543881

**Published:** 2022-07-04

**Authors:** Kanchan Bhardwaj, Agnieszka Najda, Ruchi Sharma, Renata Nurzyńska-Wierdak, Daljeet Singh Dhanjal, Rohit Sharma, Sivakumar Manickam, Atul Kabra, Kamil Kuča, Prerna Bhardwaj

**Affiliations:** ^1^School of Biological and Environmental Sciences, Shoolini University of Biotechnology and Management Sciences, Solan 173229, India; ^2^Department of Vegetable and Herbal Crops, University of Life Sciences in Lublin, 50A Doświadczalna Street, 20-280 Lublin, Poland; ^3^School of Bioengineering and Food Technology, Shoolini University of Biotechnology and Management Sciences, Solan 173229, India; ^4^School of Bioengineering and Biosciences, Lovely Professional University, Phagwara 144411, India; ^5^Department of Rasashastra and Bhaishajya Kalpana, Faculty of Ayurveda, Institute of Medical Sciences, Banaras Hindu University, Varanasi 221005, Uttar Pradesh, India; ^6^Petroleum and Chemical Engineering, Faculty of Engineering, Universiti Teknologi Brunei, Bandar Seri Begawan BE1410, Brunei Darussalam; ^7^University Institute of Pharma Sciences, Chandigarh University, Gharuan, Mohali 140413, India; ^8^Department of Chemistry, Faculty of Science, University of Hradec Kralove, 50003 Hradec Kralove, Czech Republic; ^9^Biomedical Research Center, University Hospital Hradec Kralove, Hradec Kralove, Czech Republic

## Abstract

Fresh fruit and vegetables are highly utilized commodities by health-conscious consumers and represent a prominent segment in the functional and nutritional food sector. However, food processing is causing significant loss of nutritional components, and the generation of waste is creating serious economic and environmental problems. Fruit and vegetables encompass husk, peels, pods, pomace, seeds, and stems, which are usually discarded, despite being known to contain potentially beneficial compounds, such as carotenoids, dietary fibers, enzymes, and polyphenols. The emerging interest in the food industry in the nutritional and biofunctional constituents of polyphenols has prompted the utilization of fruit and vegetable waste for developing enriched and functional foods, with applications in the pharmaceutical industry. Moreover, the utilization of waste for developing diverse and crucial bioactive commodities is a fundamental step in sustainable development. Furthermore, it provides evidence regarding the applicability of fruit and vegetable waste in different food formulations especially bakery, jam, and meat based products.

## 1. Introduction

In Europe, approximately 89 million tons of food waste is produced per year, and this amount is predicted to increase by 40 times in forthcoming years. Approximately 40% of food that is produced in India is wasted, as stated in the Food and Agriculture Organization (FAO) report [1, 2]. In addition, the Food Corporation of India reported a reduction of approximately 10–15% in the total food production. According to the Ministry of Food Processing Industries (MFPI), India calculated the total fruit and vegetable losses at approximately 12 and 21 million tons, respectively. This amounts to approximately USD 4.4 billion in value, with a total value of waste and loss of food reaching USD 10.6 billion [1]. The term “fruit and vegetable waste” (FVW) is defined as an indigestible part of the produce that is thrown away at a certain point, for example, during handling, collection, processing, and shipping [3]. According to this definition, FVW can be understood as a loss of fruit and vegetables, rather than waste. FVW can be generated during various stages, from the fields to the consumer, including both the pre- and postconsumer stages of the food supply chain [4]. FVW contains high amounts of phytochemical constituents and is studied for phenolic compounds, dietary fibers, and relative extraction of bioactive compounds [5]. Studies have reported that many essential nutrients, bioactive compounds, and other elements are abundantly found in seeds, peels, fruits, and vegetables [1]. For example, the skin of some fruits, such as lemons, avocados, seeds, grapes, mangoes, and jackfruits, contains 15% more phenolic compound concentrations than pulp fruit [6, 7]. The FVW can be used to obtain natural compounds that are useful in the textile, food, cosmetics, and pharmaceutical industries. Much of the FVW that is generated from horticultural crop supplies is now considered to be unnecessary. Their appropriate use will help resolve environmental issues and will function as a sustainable approach to improve health using enriched foods, including health-improving substances [8].

**Figure 1:**
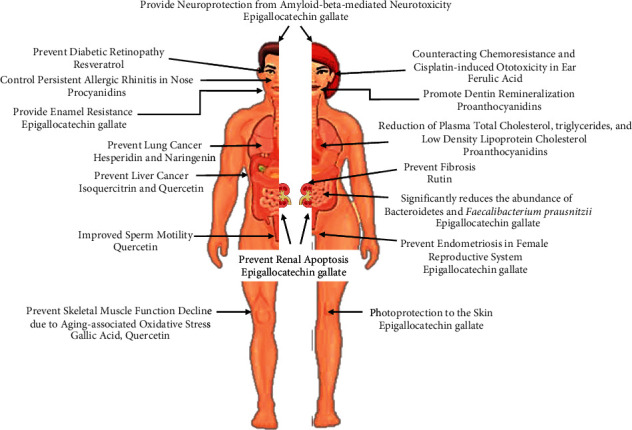
A brief depiction of the role of polyphenols in human health, based on a literature survey.

Plants produce many secondary metabolites that offer different functions, including defending themselves from microbial and pest attacks, helping them to adapt to different types of harsh environments, and providing them with the potential to resist biotic and abiotic stresses. In a wide range of natural plant metabolites, phenolic compounds have recently gained significant attention, due to their anticlotting, antimutagenic, anti-inflammatory, antidiabetic, and antioxidant properties, which are related to their ability to decrease cancer development and the risk of developing cardiovascular diseases (Figure 1) [9–12]. Polyphenolic compounds are considered as primary dietary sources that are present in fruits [13]. Previous studies revealed that the powders and extracts of fruit juices demonstrated various types of biological activities. They can be helpful as functional ingredients in dairy food industries [14, 15]. However, fruit and vegetable production is affected by seasonal variation, and the excess demand for fresh fruit and vegetables in the market has led researchers to explore alternative sources and strategies for the bioproduction of naturally occurring phenolic acids and anthocyanins compounds [16]. For example, plant callus and in vitro cell cultures were considered as promising tools for polyphenolic, anthocyanin, and other compounds produced in cherries, carrots, and grapes [16–18]. Additionally, these in vitro cultures exhibited different advantages over fresh fruit extracts in the possibility of continuously forming bioactive compounds at a large scale, depending on the general needs, with a lower cost and the opportunity of changing the anthocyanin, phenolic, or other biosynthesis pathways [19, 20]. Fruit and vegetables are common sources of natural compounds; however, at the time of processing, many byproducts (such as cores, seeds, peels, and pomaces) are disposed of, reaching between 25% and 30% of waste for the entire commodity group [8, 21]. These byproducts show an unusual, or higher, amount of phytochemical compounds compared to the traditionally exploited vegetable part; thus, they can be utilized for food product formulation and fortification [22]. The developing countries use FVW, especially peels, for synthesizing valuable products like carbon dots, biochar, biosorbents, and edible films which make them sustainable as well as eco-friendly products that can used for useful purposes. But all these interventions are still in their infancy stage and demand more exploration and advance in findings. The potential application makes them emerging topic for exploring the potential of using FVW. Therefore, the current review focuses on the different aspects of fruit and vegetable peel waste and sheds light on the availability of bioactive compounds in waste. It also discusses the potential usage of the byproducts of food processing waste, particularly in the context of peels, in both the nutraceutical and functional food industries to produce value added products.

## 2. Fruit and Vegetable Peels as a Rich Source of Polyphenols and Dietary Fiber

Bioactive plant compounds, including phenols, are responsible for the nutritional quality and sensory characteristics of fruits and vegetables [8, 23], among other functions (Table 1). These polyphenolic compounds are considered to be the largest bioactive compound groups with diverse vital biological functions [39, 40]. These compounds contain one or more aromatic ring structures, along with one or more alcoholic groups in their structure [41] that have antioxidant potential (Table 2) [56]. These compounds are different in many classes, such as flavonoids (subclasses: anthocyanidins, flavanones, flavanonols, flavonols, isoflavones, and flavones), lignans, phenolic acids, tannins, and stilbenes [57, 58].

Munir et al. [59] state that the methanol and ethanol extracts of garlic, onion, and cauliflower waste show antioxidant potential. Total phenolic contents of these vegetable waste extracts were in the range of 2.23–16.12 mg gallic acid equivalents/gram (GAE/g) of dry weight (DW), while total flavonoids were in the order of 0.24–2.13 mg catechin equivalent/gram (CE/g) of DW. This study shows that maximum inhibition capacity and maximum scavenging activity were displayed by ethanolic extract of onion waste.

The citrus industry generates seed or peel residues in high amounts, accounting for 50% of the whole fruit [39, 60]. Citrus waste is a high source of polyphenolic compounds because the peel of citrus fruits contains a larger amount of polyphenols, compared to the edible portion of the fruit [41]. Montenegro-Landívar et al. [37] proved that the orange and spinach residues are rich in polyphenols (0.51 ± 0.02 mg GAE/g fw and 0.47 ± 0.03 mg GAE/g fw, respectively) and have high antioxidant activity (2.27 mg TE g-1 and 0.04 mg TE g-1, respectively) in comparison with carrot, celery, kiwi, strawberry, and broccoli and lower antioxidant activity than kale and white and red grape. Suleria et al. [61] found that fruit peels have a diverse range of phytochemicals: the mango peel exhibited the highest phenolic content for TPC (27.51 ± 0.63 mg GAE/g) and TFC (1.75 ± 0.08 mg QE/g), while the TTC (9.01 ± 0.20 mg CE/g) was slightly higher in the avocado peel than in the mango peel (8.99 ± 0.13 mg CE/g). Moreover, grapefruit peel had the highest radical scavenging capacities for the DPPH and ABTS, ferric reducing capacity in FRAP, and total antioxidant capacity, compared to other fruit peel samples. Apart from fruit citrus, the peels from other fruits have also been reported to contain a high quantity of phenolic compounds, compared to the edible parts. Gorinstein et al. [6] found that the total phenolic content in the peel of pears, peaches, and apples was double the amount in the peeled fruits. Other studies revealed that banana (*Musa* Cavendish) pulp constituted 232 mg/100 g DM of phenolic compounds, with approximately 25% being present in the fruit peel [62]. Along with polyphenolic compounds, dopamine, L-DOPA, and catecholamines were also reported in high amounts in the peel of bananas [43]. In 2002, the National Academy of Science defined dietary fiber as a complex including lignin and nondigestible essential carbohydrates that keeps the plants intact. Functional fibers constitute isolated, nondigestible carbohydrates, which have beneficial physiological effects for human beings, in terms of total fiber (active fiber and dietary fiber) [63].

Stojanović et al. [64] reported in their study that aqueous-ethanolic pomegranate peel extract (PPE) contains maximum amount of ellagic acid, punicalagin, and punicalin. They also revealed that PPE has efficiency in improving the health of type 1 diabetes induced mice C57BL/6 by inhibiting immune cell infiltration into pancreatic islets and through interference with production of IFN-*γ* and IL-17 in gut-associated lymphoid tissue (GALT). Parkar and Addepalli [65] revealed that alcoholic OPE of 200 mg/Kg has the ability to improve the diabetic nephropathy in rats. Jung et al. [10] argued that onion peel quercetin derivatives are considered as important flavonoids for treating diabetes as they can improve diabetic status in animal and cell models. They found that oral administration of 1% onion peel extract (OPE) significantly improved the glucose tolerance and raised liver glycogen level and skeletal muscles of diabetic male Sprague-Dawley rats. The study revealed that OPE is highly efficient in glucose response and insulin resistance associated with type 2 diabetes by upregulating glucose uptake at peripheral tissues and/or downregulating inflammatory expression of gene in liver, in addition to improving free fatty acid metabolic dysregulation and reducing the oxidative stress.

Dietary fibers are present in every onion layer, although not in similar ratios. Jaime et al. [66] reported that the whole onion, from the outer skin to the inner layers, contains three diverse varieties of dietary fiber. Out of the total dietary fibers (TDFs) of the “Grano de Oro” onion, the inner layer included the lowest amount (11.6%) of dry matter (DM), while the skin contained the highest amount (68.3% DM). Ncobela et al. [67] study revealed that potato peel contains 61.0 to 125 g/kg crude fiber in the DM. The peels of fresh carrots were reported to contain dietary fiber, the amount of which increased with blanching. After blanching, the TDF content of the peels rose significantly from 45.45% DM to 73.32% DM [50].

The dietary fiber content in apple peel is higher than it is in the pulp (0.91% fresh weight (FW)). The percentage of soluble and insoluble dietary fibers was 0.43% FW and 0.46% FW, respectively [6]. The peels of mangoes included 51.2% DM TDFs (19% DM soluble fibers and 32% DM insoluble fibers) [68, 69]. Ajila and Prasada Rao [70] analyzed the dietary fiber of mango peels and revealed that the content of TDF was between 40.6% and 72.5%, and arabinose, glucose, and galactose were the neutral sugars in both soluble and insoluble dietary fibers. The “Liucheng” orange peel included approximately 57% DW TDFs, of which the soluble fraction was 9.41% DW and the insoluble fraction was 47.6% DW. The components of the primary fiber were characterized as pectin polysaccharides and cellulose [71]. The dietary fiber content of lemon peels was approximately 14 g/100 g DM, which was more than that of peeled lemon (7.34 g/100 g DM) [72]. The total soluble and insoluble dietary fibers were 4.93 g/100 g DM and 9.04 g/100 g DM, respectively. Peeled peaches included 17.0 g/100 g hemicellulose and 13.1 g/100 g alcohol insoluble residue (AIR) cellulose, whereas the unpeeled peaches held 16.4 g/100 g hemicellulose and 12.9 g/100 g AIR [73].

## 3. Fruit and Vegetable Peel-Enriched Ready-to-Eat and Ready-to-Cook Food

**Figure 2:**
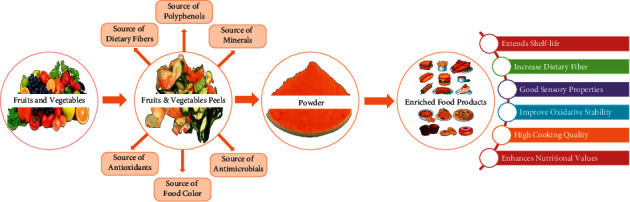
Various effects of peel enrichment on the quality of food.

There is substantial information available in the published literature about FVW as a rich source of diverse phytochemicals, particularly polyphenols with biological potential [74]. Therefore, different ready-to-eat and ready-to-cook food products, developed using fruit and vegetable peels, are listed in Table 3, and Figure 2 highlights the influence of peels on the quality of food attributes.

### 3.1. Peel Enrichment of the Physicochemical Properties of Food

Dhingra et al. [89] incorporated potato peel fiber in biscuits at concentrations of 0%, 5%, 10%, and 15%. The results showed an increase in fat, carbohydrate, and ash content. In addition, the incorporation of apple peel powder in pasta resulted in an increment in the total polyphenol content and antioxidant activity [91]. The use of watermelon rind and Sharlyn melon peel powder at the levels of 5%, 10%, and 15% increased the moisture content of cake from the initial 27.09% to 24.12% and 25.89%. This shows that the substitute materials maintained the moisture content of cakes during storage at all levels [28].

### 3.2. Peel Enrichment of the Texture Properties of Food

Texture is a crucial feature of food quality. The hardness, cohesion, and water content are the most frequently mentioned characteristics of textures [92]. In a study carried out by Goda et al. [80], the results indicated that the extruded snack foods that were fortified with 0.60% mango peel had a hardness value of 26.3 N. Iuga and Mironeasa [93] reported that the dough cohesiveness and the fracturability of the pasta were higher compared to the control, while the chewiness was lower. Gaita et al. [94] reported a grainy texture of pasta when grape pomace was added at a concentration of 3%. According to Younis et al. [95], the firmness and chewiness of a jam that was combined with mosambi peel powder increased significantly, compared to the control. However, the adhesiveness and cohesiveness of the jam decreased as the amount of mosambi peel powder increased. Similarly, the addition of orange peel at a concentration of 12% reduced the adhesiveness of the orange jam [96].

### 3.3. Peel Enrichment of the Appearance of Food

The surface color of the dried noodles with 3–9% pitaya peel powder ranged from pink to red-purple, whereas the color of the cooked noodles varied between pale yellow and orange-yellow. The cooked noodles with pitaya peel powder had much more yellowness than the control noodles [97]. Jam that was prepared by adding mosambi peel powder was highly acceptable when the powder was added at a level of 5% [95].

### 3.4. Peel Enrichment of the Microbiological Quality of Food

The use of fruit and vegetable byproducts provides the product with higher oxidative stability and a longer shelf life and minimizes microbial decomposition. The recovery of these bioactive compounds from the food industry has also been a new trend for the production of high-value bread goods that are sustainable [98]. Garcia et al. [99] reported the absence of *Salmonella* and coagulase-positive *Staphylococcus* in cookies that were incorporated within passion fruit peel flour. When incorporated into pasta, the microbial analysis of ridge gourd peel powder showed no microbial growth when stored for 15 days at room temperature [100].

### 3.5. Peel Enrichment of the Sensory Attributes of Food

According to the study carried out by Shiau et al. [97], the addition of 3–6% pitaya peel powder into noodles showed the highest acceptable sensory quality. The addition of up to 8% orange peel in a jam maintained its sensory acceptability [96].

## 4. Fruit and Vegetable Peel-Enriched Muscle Foods

Muscle food products (fish and meat) are rich sources of valuable nutrients; however, they are primarily prone to microbial contamination, protein decomposition, and lipid oxidation, which results in rapid spoilage [101]. The oxidative changes of muscle foods not only cause the aggregation of toxic compounds, but also result in undesirable changes in the flavor, color, and texture and reduce the acceptability and shelf life of the product [102]. Therefore, to inhibit these undesirable changes, different muscle food products have been developed using fruit and vegetable peels, as listed in Table 4.

### 4.1. Peel Enrichment of the Physicochemical Properties of Muscle Foods

Qin et al. [119] reported that the use of pomegranate rind powder extract in ground pork meat, at a dose of 0.02 g extract/100 g meat, resulted in the retardation of lipid oxidation. The use of banana peel powder in chicken sausages increased their nutritional value, dietary fiber content, cooking yield, and water-holding capacity, while reducing their fat content. Moreover, sweet potato peel flour with high fiber content modified the texture of dough, making it more brittle when it was added to the hamburger [120].

### 4.2. Peel Enrichment of the Texture Properties of Muscle Foods

Texture affects the technological aspects of meat products and significantly impacts consumer satisfaction [121]. A substantial increase in gumminess and chewiness was observed when dried carrot pomace, at a level of 9%, was added to sausages [122]. García et al. [118] observed that the addition of dry tomato peel at a 4.5% concentration resulted in the modification of textural properties, due to the presence of fiber.

### 4.3. Peel Enrichment of the Appearance of Muscle Foods

The color of meat and meat products is an essential indicator of freshness. Natural antioxidants have been shown to extend the color stability of meat products by inhibiting lipid oxidation and preventing the transformation of meat metmyoglobin to oxymyoglobin [111]. Zaini et al. [123] incorporated banana peel powder into fish patties, and this increased their redness value (*a*^*∗*^) and decreased their yellowness value (*b*^*∗*^). Hartmann et al. [124] incorporated pumpkin peel flour at a 3-4% concentration in bovine burgers and reported an increase in the *L*^*∗*^, *a*^*∗*^, and *b*^*∗*^ values.

### 4.4. Peel Enrichment of the Microbiological Quality of Muscle Foods

Beef burgers containing different ratios of pomegranate peel powder (1%, 2%, and 3%) exhibited a progressive reduction in total bacterial counts (2.87, 2.41, and 2.18 log cfu/g, respectively) from the initial count of 3.32–3.66 log cfu/g [113]. Hafez and Eissawy [107] reported antimicrobial activity of banana peel extract against Staphylococcus aureus, aerobic microorganisms, and Enterobacteriaceae in the marinated beef as marination ingredients. Salmonella spp. and E. coli were found to be absent from beef samples. As a result, it can be utilised as a natural meat preservative. As a result, banana peel extract can be utilized as a natural meat preservative.

### 4.5. Peel Enrichment of the Sensory Attributes of Muscle Foods

The sensory evaluation of meat is a systematic and valuable approach for determining the quality of processed meat products, particularly following the addition of a new ingredient [107]. In this respect, Fourati et al. [125] investigated the impact of pomegranate peel ethanol extract, at different concentrations, on the sensory attributes of minced beef. They reported higher acceptability when it was used at a concentration of 1%. Similarly, the addition of tomato peel at a 3% level did not affect the acceptability of sausages [126].

## 5. Conclusion and Future Perspectives

In current scenario, management of FVW has become primarily important for sustainable development. Thus, it has become a dire need to explore alternative solutions to make full use of FVW to gain the economic, environmental, and social benefits from these waste materials. In this regard, the above comprehensive content covers the literature about the potential utilization of FVW in developing innovative and healthy ready-to-eat, ready-to-cook, and muscle foods. FVW especially peel have accorded to contain sustainable bioactive compounds, with a wide range of biological potential and nutritional values that can be used for developing healthy food products. This is a step toward waste reduction in the food chain and a new way to develop diversified and innovative food products, creating a market for sustainable and functional products. Thus, it has become crucial for sensory scientists, food technologists, and nutritionists to collaborate and face the challenge of formulating more well-accepted and palatable foods. Furthermore, efforts must be made to understand the potential food safety concern, as well as consumers' perceptions of utilizing FVW in food production and formulation. Moreover, the utilization of peel-enriched probiotic drinks and yogurt is another option where food industries explore the synergism between plants and microbes to improve the human gut health. Apart from this, utilization of these FVW byproducts in food industry will enable us to develop valuable alternatives to functional food with low cost for consumers.

## Figures and Tables

**Table 1:** Polyphenolic compounds in selected fruit and vegetable peels.

Scientific name	Common name	Compounds	References
*Malus* 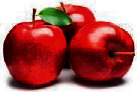	Apple	Caffeic acid, caffeic acid-4-O-glucoside, 5-caffeoylquinic acid, 3-caffeoylquinic acid, 3,4-dicaffeoylquinic acid, petunidin 3-O-(6′-*p*-coumaroyl)-glucoside), malvidin 3-O-(6′-*p*-coumaroyl)-glucoside, cyanidin 3-O-arabinoside, peonidin 3-O-glucoside, malvidin 3-O-glucoside, epicatechin, catechin 3-O-glucose, rutin, quercitrin, quercetin, phloridzin, phloretin	Călinoiu et al. [24]
*Musa* × *paradisiaca* 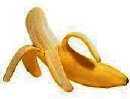	Banana	Gallocatechin, epigallocatechin, epigallocatechin gallate	Chueh et al. [25]
*Citrus* × *sinensis* 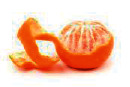	Orange	Catechin, caffeic acid, naringin, epicatechin, rutin, quercitrin, quercetin, kaempferol, luteolin	Omoba et al. [26]
*Citrus reticulate* 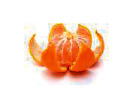	Kinnow	Gallic acid, chlorogenic acid, ferulic acid, coumaric acid, caffeic acid, catechins, epicatechins, hesperidin, naringenin, quercetin, kaempferol	Safdar et al. [27]
*Cucumis melo* 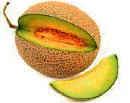	Melon	4-Hydroxybenzoic acid, vanillin, chlorogenic acid, coumaric acid	Al-Sayed et al. [28]
*Mangifera indica* 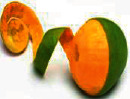	Mango	(+)-Catechin, (-)-epicatechin, (-)-epicatechin gallate, (-)-epigallocatechin gallate, procyanidin A_2_, procyanidin B_1_, procyanidin B_2_, kaempferol 3-glucoside, myricetin, isorhamnetin, rutin, quercetin 3-glucoside, quercetin-3-o-glucopyranoside, trans-resveratrol, gallic acid, cinnamic acid, *p*-coumaric acid, *o*-coumaric acid, benzoic acid, syringic acid	Coelho et al. [29]
*Citrus maxima* 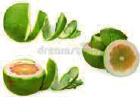	Pomelo	Naringenin, hesperetin, eriodictyol, eriocitrin, narirutin, naringin, hesperidin, neohesperidin, neoeriocitrin, neoponcirin, luteolin, diosmetin, rhoifolin, diosmin, neodiosmin, lucinen-2, vicenin-2, apigenin 6-C-glucosyl-7-O-glucoside, diosmetin 6,8,-di-C-glucoside, diosmetin 6-C-glucoside, rutin, quercetin, kaempferol	Tocmo et al. [30]
*Punica granatum* 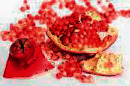	Pomegranate	Punicalagin, granatin B, tellimagrandin I, casuarinin, granatin A, pedunculagin, punicalin, corilagin, gallagic acid, ellagic acid, caffeic acid, catechin, gallocatechin, luteolin, kaempferol, *p*-coumaric acid, gallic acid,	Akhtar et al. [31]
*Actinidia deliciosa* 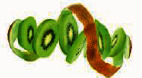	Kiwifruit	Protocatechuic acid, caffeic acid, chlorogenic acid, quinic acid, (+)-gallocatechin, proanthocyanidin B_2_, proanthocyanidin C_1_, quercetin 3-glucoside, quercetin 3-O-rutinoside, quercetin 3-O-galactoside	Zhang et al. [32]
*Daucus carota* subsp. *sativus* 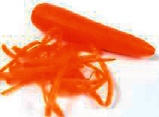	Carrot	Chlorogenic acid	Zhang and Hamauzu [33]
*Allium cepa* 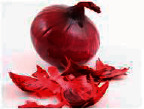	Onion	Protocatechuic acid, quercetin 3,4′-diglucoside, quercetin-3-glucoside, quercetin, kaempferol	Celano et al. [34]
*Allium sativum* 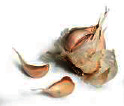	Garlic	Caffeic acid, p-coumaric, ferulic, and di-ferulic acids	Kallel et al. [35]
*Solanum tuberosum* 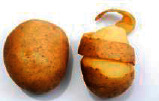	Potato	Caffeic acid, chlorogenic acid, ferulic acid, gallic acid, protocatechuic acid	Singh et al. [36]
*Spinacia oleracea* 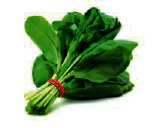	Spinach	Caffeic acid, ferulic acid, rutin	Montenegro-Landívar et al. [37]
*Solanum lycopersicum* 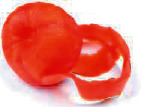	Tomato	Caffeic acid, procatchoic acid, vanillic acid, catechin, gallic acid	Elbadrawy and Sello [38]

**Table 2:** Polyphenolic antioxidant activities of selected fruit and vegetable peels.

Scientific name	Common name	Polyphenol extraction method	Solvent used and extraction conditions	Total phenolic content	Antioxidant activities		References
*Malus domestica*	Apple	Enzymatic (cellulolytic and pectolytic)	ND, 30–50°C/12–24 h	0.38 ± 0.02 mg/mL	Antioxidant activities increased with concentration of extract in DPPH (0.10 to 0.40 vitamin C eq mM), ABTS (0.09 to 0.28 vitamin C eq mM), and FRAP (0.06 to 1.85 FeSO_4_ eq mM)		Park et al. [42]
*Musa acuminata* Colla AAA	Banana	Liquid extraction	Methanol, ethanol, acetone, water acidified with hydrochloric acid, 25°C/1 min	3.3 ± 0.8% 100 g	High capacity to scavenge DPPH and ABTS		González-montelongo et al. [43]
*Citrus reticulate*	Kinnow	Microwave-assisted extraction	Methanol, 1–120°C/999s	37,793.37 ± 52.39 *μ*g/g	High hydroxyl radical scavenging (26.03%)		Hayat et al. [44]
*Citrus unshiu* Marc.	Satsuma mandarin	Ultrasonic-assisted extraction	Methanol, for phenolic acid 30°C/20 min and for flavanone glycosides 40°C/60 min	1935.12 ± 50.52 *μ*g/g	Good antioxidant activity using FRAP		Ma et al. [45]
*Actinidia deliciosa*	Kiwifruit	Subcritical water extraction	Water, 160°C/20 min	51.2 mg GAE/g	Antioxidant activities increasing by the increase in the extraction temperature for DPPH, ABTS, and FRAP		Guthrie et al. [46]
*Mangifera indica*	Mango	Liquid extraction	Acetone, room temperature/15 min	90 to 110 mg/g (in raw) and 55 to 100 mg/g (in ripe)	Good antioxidant activity (for DPPH assay, IC_50_ values were found to be in the range of 1.39–5.24 *μ*g GAE)		Ajila et al. [47]
*Cucumis melo*	Melon	Liquid extraction	Methanol, 25°C/15 min	0.69 to 2.96 mg GAE/g	Good antioxidant activity, ranging from 0.13 to 0.26 mg ascorbic acid equivalent/mL extract using ABTS assay		Ganji et al. [48]
*Citrus grandis L*.	Pomelo	Supercritical carbon dioxide extraction	Ethanol, 80°C/49 min	ND	High capacity to scavenge hydroxyl radical, DPPH, and ABTS		He et al. [49]
*Daucus carota*	Carrot	Liquid extraction	Water, 90°C/1 min	837 ± 61 mg GAE/100 g	Total antioxidant activity of 94.67%		Chantaro et al. [50]
*Solanum melongena L*.	Eggplant	Ultrasonic-assisted extraction	2-Propanol, 60°C/40 min	7.284 g/100 g	Good antioxidant activity (5.37 *μ*M trolox/kg) using ABTS assay		Dranca and oroian [51]
*Allium sativum L*.	Garlic	Liquid extraction	Ethanol, room temperature/24 h	63.05 ± 0.20 mg CAE/g	Good antioxidant activity (DPPH assay IC_50_ values: 0.20 ± 0.00 mg/mL; ABTS IC_50_ value: 0.44 ± 0.01 mg/mL)		Kim et al. [52]
*Zingiber officinale*	Ginger	Liquid extraction	Ethanol, room temperature/5 min	52.57 ± 0.06 mg GAE/g		Scavenging activity of 4.58 ± 0.06 mg TE/g with DPPH and 47.77 ± 1.60 mg TE/g with FRAP	Tinello and Lante [53]
*Curcuma longa*	Turmeric	Liquid extraction	Ethanol, room temperature/5 min	104.88 ± 0.15 mg GAE/g		Scavenging activity was 5.55 ± 0.11 mg TE/g with DPPH and 70.73 ± 0.82 mg TE/g with FRAP	Tinello and lante [53]
*Artocarpus heterophyllus L*.	Jackfruit	Solid-liquid extraction	Methanol, room temperature/6 h	48.05 ± 4.57 mg GAE/g		Good antioxidant activity (DPPH assay IC_50_ values: 1.25 ± 0.02 mg/mL; ABTS IC_50_ value: 0.23 ± 0.02 mg/mL)	Zhang et al. [54]
*Allium cepa*	Onion	Ultrasonic-assisted extraction	Ethanol, 25°C/30 min	ND		Scavenging activity was 7.82 ± 0.72 *μ*M TE/g with DPPH and 11.32 ± 1.40 *μ*M TE/g with ORAC	Celano et al. [34]
*Raphanus sativus* L. var. *niger*	Black radish	Liquid extraction	Ethanol and water, 25°C/2 h	305.51 ± 5.2 mg GAE/g		Scavenging activity was 36% with DPPH, 6% with CUPRAC, and 47% with FRAP	Yücetepe et al. [55]

ND: not defined; GAE: gallic acid equivalents; CAE: caffeic acid equivalents; DPPH: 2,2-diphenyl-1-picrylhydrazyl; ABTS: 2,20-azino-bis(3-ethylbenzothiazoline)-6-sulfonic acid; FRAP: ferric reducing antioxidant power assay; ORAC: oxygen radical absorbance capacity; CUPRAC: cupric reducing antioxidant capacity.

**Table 3:** Application of fruit and vegetable peels in ready-to-eat and ready-to-cook products.

Scientific name	Common name	Product		Form used		Key findings	References
*Spondias dulcis*	Caja-manga	Bread		Flour		Modification of physicochemical characteristics; good sensory acceptance	Perin et al. [75]
*Citrus sinensis* L.	Orange	Bread		Powder		Modification of fiber, pectin, and polyphenol content; strengthening the dough elasticity	Han et al. [76]
*Opuntia ficus-indica*	Prickly pear	Bread		Flour		High leavening dough capacity and bread-specific volume; increased amount of total polyphenols and betalains	Parafati et al. [77]
*Artocarpus heterophyllus*	Jackfruit	Cookies		Powder		High acceptability; increase in darkness of cookies; decrease in spread ratio	Ramya et al. [78]
*Passiflora edulis*	Passion fruit	Biscuit		Flour		Higher fat absorption capacity; improvement of texture; high fiber content	Weng et al. [79]
*Mangifera indica*; *Cucurbita maxima* L.	Mango, pumpkin	Extruded snack		Powder		Highest bulk density and hardness; enhancement of antioxidant activity	Goda et al. [80]
*Syzygium cumini*	“Jamblang”	Jam		ND		Increase in total phenol and anthocyanin content	Anggraini et al. [81]
*Citrus sinensis*	Orange	Biscuits		Powder		Increase in dietary fiber; enhancement of nutritional value, physical quality, and overall acceptability	Zaker et al. [82]
*Citrullus lanatus*	Watermelon		Noodles	Powder	High nutritional value; greatest acceptability		Ho and Che Dahri [83]
*Musa acuminata*	Banana		Bread	Powder	Higher protein, carbohydrate, and fat content		Eshak [84]
*Punica granatum*	Pomegranate		Wheat noodles	Extract	Increase in DPPH radical scavenging activity; decrease in pH; alteration in color and texture; no significant difference in terms of firmness and stickiness		Kazemi et al. [85]
*Opuntia ficus-indica*	Passion fruit		Bread	Flour	Higher fiber content, ash quantity, and hardness value; lower specific volume		Conti-Silva et al. [86]
*Punica granatum*	Pomegranate		Cookies	Powder	Increase in dietary fiber, mineral content, total phenolic compounds, and antioxidant activity; reduction in oxidative degradation		Ismail et al. [87]
*Punica granatum*	Pomegranate		Biscuits	Powder	Increase in hardness and breaking strength; decrease in cohesiveness, springiness, and spread ratio		Srivastava et al. [88]
*Solanum tuberosum*	Potato		Biscuits	ND	Increase in breaking strength with increase in dietary fiber		Dhingra et al. [89]
*Mangifera indica*	Mango		Macaroni	Powder	Increase in total dietary fiber, antioxidant properties, and firmness; enhancement of nutritional quality		Ajila et al. [90]

ND: not defined.

**Table 4:** Application of fruit and vegetable peels in muscle foods.

Scientific name	Common name	Form used	Food commodity	Storage conditions	Key findings	References
*Musa balbisiana*	Banana	Powder	Chicken sausage	4°C/ND	Delay in lipid oxidation; increase in storage modulus values	Zaini et al. [103]
*Punica granatum* L.	Pomegranate	Powder	Chicken meat patty	−18 ± 2°C/ND	Higher total phenolic content; higher water-holding capacity, ash, crude fiber content, and hardness values; lower moisture content and lightness values	Sharma and Yadav [104]
*Allium cepa* L.	Onion	Powder	Sausage	5°C/28 days	Increase in antioxidant activity and total polyphenol content; decrease in pH	Bedrníček et al. [105]
*Solanum lycopersicum* L.; *Punica granatum* L.	Tomato and pomegranate	Powder	Sausage	−18°C/4 months	Improved sensorial characteristics, water-holding capacity; lower cooking loss values	Hussien et al. [106]
*Musa acuminata*	Banana	Extract	Marinated beef	4°C/4 hours	Good sensory properties; antibacterial activity against the aerobic colonies, *Enterobacteriaceae* and *Staphylococcus aureus*	Hafez and Eissawy [107]
*Prunus salicina*	Plum	Microparticles	Breast chicken patty	4°C/10 days	Higher cyanidin content providing intense fiber's red color	Basanta et al. [108]
*Punica granatum L*.	Pomegranate	Nanoparticles	Meatballs	4°C/15 days	Improvement in cooking characteristics during storage; lower microbial load; retarded lipid oxidation	Morsy et al. [109]
*Musa paradisiaca* L. cv. Dominico Harton	Plantain	Flour	Frankfurter-type sausage	4°C/48 hours	Increase in water retention capacity	Rosero-Chasoy and Serna-Cock [110]
*Citrus sinensis*	Orange	Powder	Beef burger	−18°C/ND	Improvement in cooking properties; increase in phenolic content; retardation of lipid oxidation	Mahmoud et al. [111]
*Ananas comosus*	Pineapple	ND	Beef burger	−18°C/ND	Reduction in cooking loss; increase in hardness	Selani et al. [112]
*Punica granatum* L.	Pomegranate	Powder	Beef burger	4 ± 1°C/12 days	High storage stability during refrigerated storage; improvement in microbiological criteria; high cooking quality and sensory characteristics	Abdel Fattah et al. [113]
*Punica granatum* L.	Pomegranate	Powder	Beef sausage	4°C/12 days	High storage stability; high cooking quality and sensory characteristics	El-Nashi et al. [114]
*Plinia jaboticaba (Vell.) Berg.*	Jabuticaba	Extract	Bologna-type sausage	4°C/35 days	Improvement in oxidative stability and sensory properties	de Almeida et al. [115]
*Allium cepa L*.	Onion	Extract	Raw ground pork	4°C/16 days	Decrease in pH of the samples during storage; inhibition of lipid oxidation	Shim et al. [116]
*Persea americana* Mill.	Avocado	Extract	Raw porcine patty	4°C/15 days	Reduction in the loss of redness; reduction in the increase in lightness during storage	Rodríguez-Carpena et al. [117]
*Solanum lycopersicum* L.	Tomato	Dry	Beef burger	ND	Good overall acceptability; modification of textural properties	García et al. [118]

ND: not defined.

## Data Availability

The data used to support the findings of this study are available from the corresponding authors from request.
